# Bisphenol F Drives Endoplasmic Reticulum Stress-Mediated Macrophage Polarization, Leading to Inflammation and Fibrosis in Mouse Kidneys

**DOI:** 10.3390/toxics14030255

**Published:** 2026-03-13

**Authors:** Chenjiao Miao, Yang Fu, Binwen Zhang, Wangyong Yu, Miao Song, Yanfei Li, Zheng Cao

**Affiliations:** Heilongjiang Key Laboratory for Laboratory Animals and Comparative Medicine, College of Veterinary Medicine, Northeast Agricultural University, No. 600, Changjiang Road, Harbin 150030, China; b240601017@neau.edu.cn (C.M.); fuyang@hezevc.edu.cn (Y.F.); s230601040@neau.edu.cn (B.Z.); s230601041@neau.edu.cn (W.Y.); songmiao@neau.edu.cn (M.S.)

**Keywords:** Bisphenol F, renal, fibrosis, inflammation, endoplasmic reticulum stress, macrophage polarization

## Abstract

Bisphenol F (BPF) is a chemical compound that has found extensive application in the field of plastics manufacturing. BPF exposure leads to renal dysfunction; however, the mechanism is unclear. This study investigated BPF-induced nephrotoxicity using 50 male Kunming mice divided into five groups: control (C), low-dose (L, 0.5 mg/kg), medium-dose (M, 5 mg/kg), high-dose (H, 50 mg/kg) BPF, and an intervention group receiving 4-phenylbutyric acid (4-PBA) plus BPF. Treatments were administered daily by oral gavage for 28 days. Renal function was assessed via serum creatinine (SCr), while inflammation and fibrosis were evaluated using histology, immunohistochemistry, immunofluorescence, ELISA, qRT-PCR, and Western blotting. Preliminary results suggest that BPF causes structural damage and dysfunction in the mice kidney. Furthermore, BPF-induced renal inflammation and fibrosis, accompanied by the activation of endoplasmic reticulum (ER) stress and the polarization of renal macrophages toward M1 and M2 types. In vitro, BPF (40 µM, 48 h) induced similar effects in Raw264.7 cells, which were mitigated by 4-PBA pretreatment. Finally, 4-PBA intervention confirmed that BPF triggers macrophage polarization via ER stress, leading to inflammation and fibrosis, ultimately causing renal dysfunction in vivo. This study provides new insights into BPF nephrotoxicity and a basis for therapeutic strategies.

## 1. Introduction

Bisphenol A (BPA), a widely used organic compound, is primarily used in the manufacture of polycarbonate plastics and epoxy resins, which are raw materials for products commonly used in various fields [1,2]. As an endocrine disruptor, BPA has long been demonstrated to induce multi-organ toxic responses, including in the testes, ovaries, kidneys, liver, brain, and adipose tissue [3,4]. Because of the potential risk to human health from exposure to BPA, numerous countries or regions have banned or restricted the use of this chemical [5,6,7]. Bisphenol F (BPF) was developed as the BPA alternative and is used in a variety of products. It is reported that the annual import of BPF in the European Economic Area reaches 10,000 tons [8]. As a result, BPF was detected in a variety of environmental matrices. It was also detected in human samples. This poses an ecological risk. It is even higher than that of BPA [9]. In the study of the Taihu Lake basin, BPF was exposed in the aqueous environment at concentrations as high as 445.5 ng/L [10]. A study of women’s undergarments manufactured in China revealed BPF concentrations ranging up to 45,205 ng/g [11]. The entry of BPF into the human body occurs through the ingestion of contaminated food or water or by exposure to the surrounding environmental media. From 2000 to 2014, there was a decrease in urinary BPA levels in the general population, while there was an increase in urinary BPF levels [12]. Human blood levels of BPF are three times higher than those of BPA [13]. The presence of BPF was detected in 55% of 100 urine samples from non-occupationally exposed adults in the U.S., and concentrations reached as high as 212 ng/mL [14]. In addition, the detection rate of urinary BPF in the non-occupational population in Taiwan is even as high as 100% [15]. Currently, the maximum dose of urinary BPF in humans can even reach 807 ng/mL [16]. Because of its similar chemical structure to BPA, BPF has also been suggested to potentially have toxic effects similar to those of BPA [17]. It has been demonstrated that BPF can cause toxic effects in various organs, such as ovaries [18], testes [19,20], lungs [21], bones [22], and intestines [23]. These findings underscore the critical environmental and public health implications of BPF contamination, a phenomenon that has been exacerbated by the widespread regulation of BPA.

Recently, a series of tests have identified that BPF can accumulate in the kidney [24], leading to elevated serum uric acid and urinary proteins [25], and a significant association with kidney disease [26,27]. This suggests that BPF exposure leads to kidney injury. However, the toxicological mechanism of BPF-induced renal injury remains to be further explored. In the present study, molecular docking analysis predicted potential interactions between BPF and key endoplasmic reticulum (ER) stress response proteins, leading us to hypothesize that ER stress may mediate BPF-induced nephrotoxicity. To test this hypothesis, we established in vivo and in vitro models of BPF exposure, as well as a pharmacological intervention model using the ER stress inhibitor 4-phenylbutyric acid (4-PBA). We examined renal function, inflammation, fibrosis, macrophage polarization, and ER stress signaling to investigate the potential mechanisms underlying BPF-associated renal injury. This study aims to provide new insights into the nephrotoxicity of BPF and to offer a theoretical basis for the prevention and treatment of BPF-related renal diseases.

## 2. Materials and Methods

### 2.1. Animal Treatment

A total of 50 Kunming mice (male, 35 days old, Changsheng Biotechnology Co., Ltd., Shenyang, China) were pre-housed for one week and divided equally into the control group (C), low-dose BPF exposure group (L, 0.5 mg/kg B.W. BPF), medium-dose BPF-exposed group (M, 5 mg/kg B.W. BPF), high-dose BPF exposure group (H, 50 mg/kg B.W. BPF) [20] and ER stress inhibitor 4-PBA + BPF exposure group (4-PBA+BPF, 100 mg/kg B.W. 4-PBA+5 mg/kg B.W. BPF) [28]. The sample size was chosen based on previous similar studies in the field [19], which used comparable animal numbers and successfully detected significant differences in key endpoints, and also considered practical constraints such as animal availability and ethical guidelines. BPF (Macklin, Shanghai, China) and 4-PBA (MCE, Monmouth Junction, NJ, USA) were first fully dissolved in DMSO, and then diluted with 0.9% sterile saline to achieve the final working concentrations. The final concentration of DMSO in all solutions was less than 0.1% (*v*/*v*). All mice were administered 0.1 mL of the drug daily via oral force-feeding according to their group assignments and identification numbers. Mice in the control group received an equivalent volume of normal saline, while the BPF-treated groups received BPF dissolved in an appropriate vehicle. The experimental protocol involved the treatment of mice for a period of 28 consecutive days. Mice were weighed weekly to adjust the BPF dose according to body weight. On the 29th day, the mice were weighed and blood samples were collected. This procedure was performed 4 h after food withdrawal. The mice were euthanized using the cervical dislocation method, and then the kidneys were collected and weighed. The experimental environment was maintained at a temperature of 24 ± 2 °C, with a humidity level of 50–60%, and under a 12/12 h light–dark cycle. The subjects had unrestricted access to food and water. Mice were fed a maintenance diet for mice and rats (Changsheng Biotechnology Co., Ltd., Shenyang, China). All of the above steps were approved by the Northeast Agricultural University Institutional Committee on Animal Care and Use (NEAUEC202303105). 

### 2.2. Serum Creatinine (SCr) Level Test

Blood samples were collected from the retro-orbital plexus into sterile tubes and allowed to clot at room temperature for 30 min. Serum was separated by centrifugation at 3000× rpm for 10 min at 4 °C using a refrigerated centrifuge (Micro21R, Thermo Fisher, Waltham, MA, USA). SCr levels were measured using a Creatinine Assay Kit (sarcosine oxidase method, Nanjing Jiancheng Bioengineering Institute, Nanjing, China) according to the manufacturer’s protocol. Briefly, serum samples (10 μL) were mixed with 200 μL of enzyme working solution in 96-well plates and incubated at 37 °C for 20 min. Absorbance was measured at 546 nm using a microplate reader (BioTek, Winooski, VT, USA). Creatinine concentrations were calculated from a standard curve generated using calibrators provided in the kit. All samples were assayed in duplicate. n = 3 mice per group were used.

### 2.3. Masson Staining

Kidney tissues were fixed using pre-cooled formaldehyde at 4 °C and samples were sent to Sevier Biotechnology Ltd. (Wuhan, China) for staining. n = 3 mice per group were used. The steps are described in Text S1.

### 2.4. Hematoxylin-Eosin (HE) Staining

After the sections were deparaffinized, the nuclei were stained with hematoxylin for 3 min. Then, they were differentiated with 1% hydrochloric acid–alcohol solution for 30 s. Next, the eosin staining solution was used for 2 min, and the color was washed off with distilled water. The sections were put into a type of clear resin and looked at under a microscope. n = 3 mice per group were used.

### 2.5. Immunohistochemistry

The kidney tissues were preserved using a special liquid called formaldehyde that was kept very cold (4 °C). Then, the samples were sent to Sevier Biotechnology Ltd. for staining. In short, after the sections were deparaffinized, they were closed for 30 min with closed serum. Then, the primary antibody was added dropwise and incubated in a wet box at 37 °C for 2 h. After being washed with PBS, the secondary antibody was added dropwise and incubated at 37 °C for 30 min. Then, after the addition of DAB color development solution, Mayer’s hematoxylin was added dropwise for 30 s. Finally, the sections were washed with distilled water and returned to blue solution, which was diffused and soaked for 1 min. Then, they were washed with water. n = 3 mice per group were used. The corresponding antibody information is shown in Table S1 of Text S2.

### 2.6. Molecular Docking

The 3D structures of target proteins were retrieved from the RCSB Protein Data Bank (https://www.rcsb.org/) using the following PDB IDs: protein kinase R-like endoplasmic reticulum kinase (PERK): 4YZY, inositol-requiring enzyme 1 (IRE1): 4PL3, and activating transcription factor 6 (ATF-6): 6H9U. The 3D structure of BPF was obtained from PubChem. The 3D structures of BPF and proteins were pre-processed for water removal, ligand removal, and hydrogen atom addition using PyMOL software (version 3.1.5.1). AutoDock Tools software (version 1.5.6) was used to prepare the receptor and ligand files and to set up the docking boxes to define the binding sites. The docking process was executed utilizing the AutoDock Vina 1.5.6 software, and the resulting data were rendered visually using PyMOL to assess the binding mode and binding affinity between BPF and PERK, IRE1, and ATF-6. Molecular docking analysis was performed on 20 April 2025.

### 2.7. Protein–Protein Interaction (PPI) Analysis

PPI networks were analyzed using the STRING database (version 12.0, https://cn.string-db.org/), accessed on 26 April 2025. The analysis encompassed proteins involved in ER stress (PERK, IRE1, ATF-6), macrophage polarization (CD68, CD206), inflammatory response (IL-6, TNF-α, IL-1β), and fibrosis (TGF-β1, α-SMA). The organism was set to “Homo sapiens”. Network construction employed an interaction threshold of 0.700 (medium confidence). Protein interactions were based on predictions and validation from prior studies. Target proteins were selected based on the documented literature and their observed roles in our preliminary experiments, prioritizing proteins with established functions in ER stress, inflammation, and fibrosis.

### 2.8. Cell Culture and Viability Assay

The Raw264.7 cells (Pricella, Wuhan, China) were resuspended in DMEM medium (Gibco, New York, NY, USA) containing 1% penicillin–streptomycin (BIOTIAN, Shanghai, China) and 10% fetal bovine serum (VivaCell, Shanghai, China) and cultured in a 37 °C incubator with 5% CO_2_, with the liquid changed every 24 h. After reaching 80–85% density, the cells were gently rinsed with medium and passaged at a density of 1:3. The Raw264.7 cell suspension was inoculated into 96-well plates for 24 h to reach the logarithmic growth phase. Cells were then treated with BPF at final concentrations of 0, 10, 20, 40, 80, 100 and 150 μM for 24 or 48 h. Next, 10% CCK-8 (Beyotime, Shanghai, China) was added, and the cell viability was detected using an enzyme marker after 30 min reaction.

### 2.9. Cell Treatment and Grouping

The Raw264.7 cell suspension was inoculated into the top chamber of a Transwell or into Petri dishes and 96-well plates for 24 h, with BPA and 4-PBA. Cells were divided into the following three groups: control (C), BPF group (40 µM BPF, 48 h) and 4-PBA+BPF group (10 µM 4-PBA pretreatment for 2 h, 10 µM 4-PBA + 40 µM PBF 48 h) [29]. All cells used in the experiments of this study were from generation 3 to generation 10.

### 2.10. ELISA

The concentrations of inflammatory cytokines in kidney tissue homogenates and Raw264.7 cell culture supernatants were quantified using commercially available ELISA kits. Specifically, IL-6, TNF-α, IL-1β, and IL-10 levels were measured using kits from JONLNBIO (Shanghai, China), and TGF-β1 levels were measured using a kit from JINGMEIBIO (Taizhou, China). All assays were performed according to the manufacturers’ protocols. Absorbance was read at 450 nm using a microplate reader. n = 3 mice per group were used. For detailed information, see Text S2.

### 2.11. Cell Co-Culture

For co-culture experiments, Raw264.7 macrophages were seeded in the upper chamber of Corning^®^ HTS Transwell^®^-24-well (New York, NY, USA) permeable nested plates. This nested system (Corning, NY, USA, Cat. No.: CLS3396-2EA) features a 0.4 μm pore size, and a polycarbonate (PC) membrane with a diameter of 6.5 mm. The membrane is TC-treated to ensure cell adhesion and is supplied in sterile, individually packaged units. After 24 h, NIH3T3 cells (Pricella, Wuhan, China) and TCMK-1 cells (Pricella, Wuhan, China) were inoculated in the bottom chamber and cultured for 24 h. The BPF- and 4-PBA-treated Raw264.7 cells were then replaced with fresh DMEM medium, and co-cultured with the NIH3T3 and TCMK-1 cells inoculated in the bottom chamber. They were co-cultured for 24 h for subsequent assays.

### 2.12. Immunofluorescence

Kidney tissues were fixed using pre-cooled formaldehyde at 4 °C and samples were sent to Sevier Biotechnology Ltd. for staining. For cells, fluorescent staining was performed using the previous method [30]. n = 3 mice per group were used. Steps are described in Text S3. The corresponding antibody information is shown in Table S1.

### 2.13. Quantitative Real-Time Polymerase Chain Reaction (qRT-PCR)

QRT-PCR was performed as described in our previous study [31], with detailed steps provided in Text S4. n = 3 mice per group were used.

### 2.14. Western Blotting

Western blotting was performed as described in our previous study [32]. Protein bands were visualized on Amersham Imager 600 (Fairfield, CT, USA) using immobilon chemiluminescent HRP substrate (Beyotime, Shanghai, China). The results were quantified by Image J 64-bit and normalized comparisons were made using β-actin. n = 3 mice per group were used. The Western blotting specific antibodies we used are shown in Table S1.

### 2.15. Statistical Analysis

The values are presented as the mean ± standard deviation (SD). For comparisons among multiple groups, one-way analysis of variance (ANOVA) was performed. If the data met the assumptions of normality and homogeneity of variance, post hoc pairwise comparisons were conducted using the least significant difference (LSD) test. If homogeneity of variance was violated, Dunnett’s T3 test was applied. For comparisons between two groups, Student’s *t*-test was used. Statistical analyses were performed using SPSS software (version 22.0, IBM Corp., New York, NY, USA). We considered *p* < 0.05 as significant and *p* < 0.01 as markedly significant.

## 3. Results

### 3.1. BPF Causes Renal Dysfunction, Fibrosis and Inflammation in Mice

**Figure 1:**
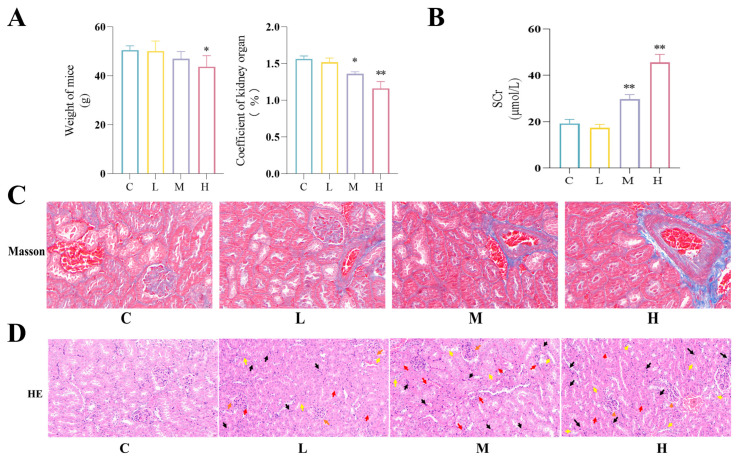
**BPF causes kidney inflammation and fibrosis in mice.** (**A**) Effect of BPF on body weight and renal organ coefficient in mice (n = 4). (**B**) Effect of BPF on SCr levels in mice (n = 3). (**C**) Effect of BPF on renal collagen fiber and collagen deposition in mice (n = 3), scale: 50 µm. (**D**) Representative HE staining images of kidney sections from one mouse per group (n = 3 per group), scale bar: 20 μm. Glomerular atrophy (orange arrow), tubular epithelial cell detachment (yellow arrow), blood exudation (red arrow), and inflammatory cell infiltration (black arrow) are indicated. * *p* < 0.05 or ** *p* < 0.01 versus C value.

To investigate the effects of BPF on the renal function, we exposed mice to different concentrations of BPF for 28 d. Results showed that moderate-dose BPF significantly reduced the renal coefficient in mice, while high-dose BPF significantly reduced body weight in mice (Figure 1A) (*p* < 0.05 or *p* < 0.01). Furthermore, we found that moderate and high doses of BPF elevated SCr levels in mice, suggesting that BPF may impair renal function. (Figure 1B) (*p* < 0.01). Masson staining results showed that BPF induced collagen and collagen fiber deposition of kidneys in mice (Figure 1C). These results indicate that BPF-induced kidney dysfunction may be associated with renal fibrosis in mice. Kidney inflammation is an important pathological factor in the development of renal fibrosis. HE results showed that BPF caused inflammatory cell infiltration, tubular epithelial cell detachment, and glomerular atrophy in renal tissues (Figure 1D). All the above results suggest that BPF-induced renal dysfunction is associated with renal fibrosis and inflammation in mice.

### 3.2. BPF Causes Renal Inflammatory Factor Expression, Macrophage Recruitment and M1/M2 Polarization, as Well as Macrophage–Myofibroblast Transformation (MMT), Epithelial–Mesenchymal Transition (EMT) and Fibroblast–Myofibroblast Transformation (FMT) in Mice

**Figure 2:**
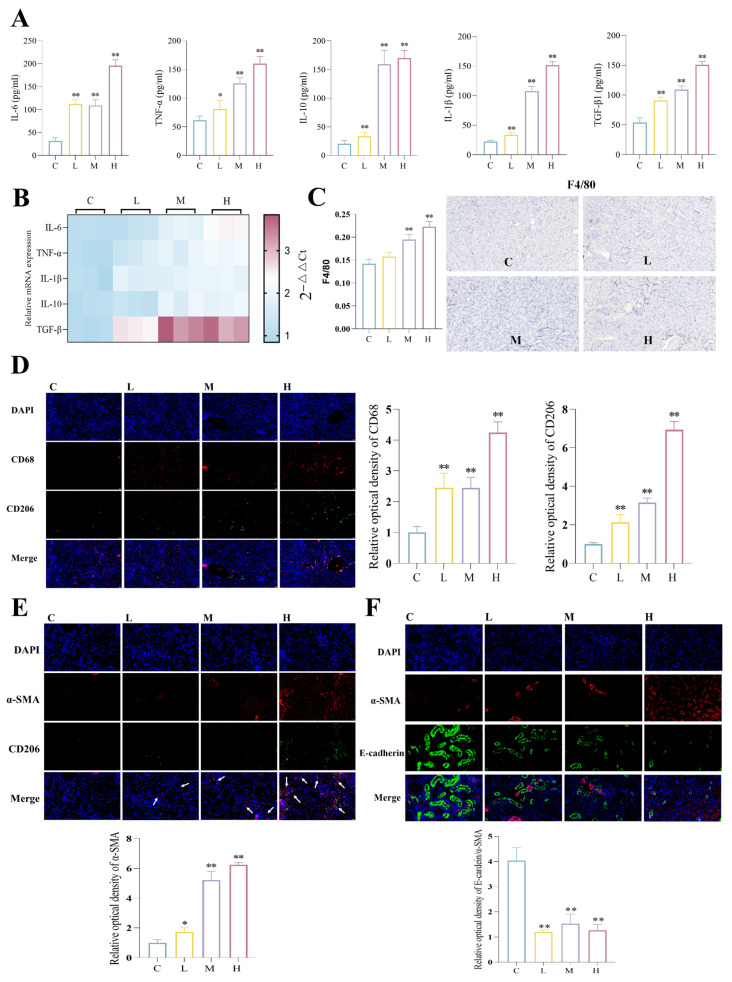
**BPF causes renal inflammatory factor secretion, macrophage recruitment and M1/M2 polarization, as well as MMT, EMT and FMT in mice.** (**A**) Effect of BPF on renal inflammatory factors in mice (n = 3). (**B**) Heatmap showing mRNA expression levels of renal inflammatory factors in BPF-exposed mice (n = 3). (**C**) BPF causes macrophage infiltration in mice kidneys (n = 3), (F4/80) scale: 50 µm. (**D**) Effect of BPF on M1/M2 polarization of kidney macrophages in mice (n = 3), scale: 50 µm. (**E**) Effect of BPF on renal MMT and FMT in mice (n = 3), scale: 20 µm. (**F**) Effect of BPF on renal tubular epithelial cell EMT in mice (n = 3), scale: 20 µm. * *p* < 0.05 or ** *p* < 0.01 versus C value.

To investigate the mechanism by which BPF causes renal inflammation and fibrosis, we examined the effect of BPF on the expression of renal inflammation-related factors. The results showed that BPF led to an increased expression of renal inflammation-associated factors mRNA and protein, including IL-6, TNF-α, IL-1β, IL-10, and TGF-β1 (Figure 2A,B) (*p* < 0.05 or *p* < 0.01). In the inflammatory response, increased expression of pro-inflammatory factors is a result of macrophage M1 polarization, whereas increased expression of anti-inflammatory factors is a result of macrophage M2 polarization. Consequently, the present study examined the effects of BPF on renal macrophages. The application of immunohistochemical labeling to macrophages revealed that BPF led to the infiltration of macrophages into the kidneys (Figure 2C) (*p* < 0.01). Meanwhile, IF results revealed that BPF significantly enhanced the fluorescence intensity of CD68 and CD206 in mouse renal macrophages (Figure 2D) (*p* < 0.05 or *p* < 0.01). Comprehensive inflammation factor indicators demonstrated that BPF induced an increase in both M1 and M2 macrophage phenotypes.

We then found that BPF resulted in enhanced fluorescence intensity of α-SMA, suggesting FMT; furthermore, renal α-SMA co-localized with CD206 was increased, suggesting that BPF resulted in mouse renal MMT (Figure 2E) (*p* < 0.01). Furthermore, immunofluorescence was utilized to visualize the expression of α-SMA and E-cadherin proteins. It was ascertained that the ratio of E-cadherin to α-SMA was diminished in renal tubular epithelial cells, thereby indicating an EMT in these cells (Figure 2F) (*p* < 0.01). In light of these observations, we hypothesize the following: BPF may promote the secretion of pro-inflammatory factors by inducing macrophage M1-type polarization to cause renal inflammation; also, it may promote the secretion of anti-inflammatory factors by inducing macrophage M2-type polarization to cause renal fibrosis by mediating the secretion of MMT, FMT, and EMT.

### 3.3. BPF Exposure Activates Renal ER Stress in Mice

**Figure 3:**
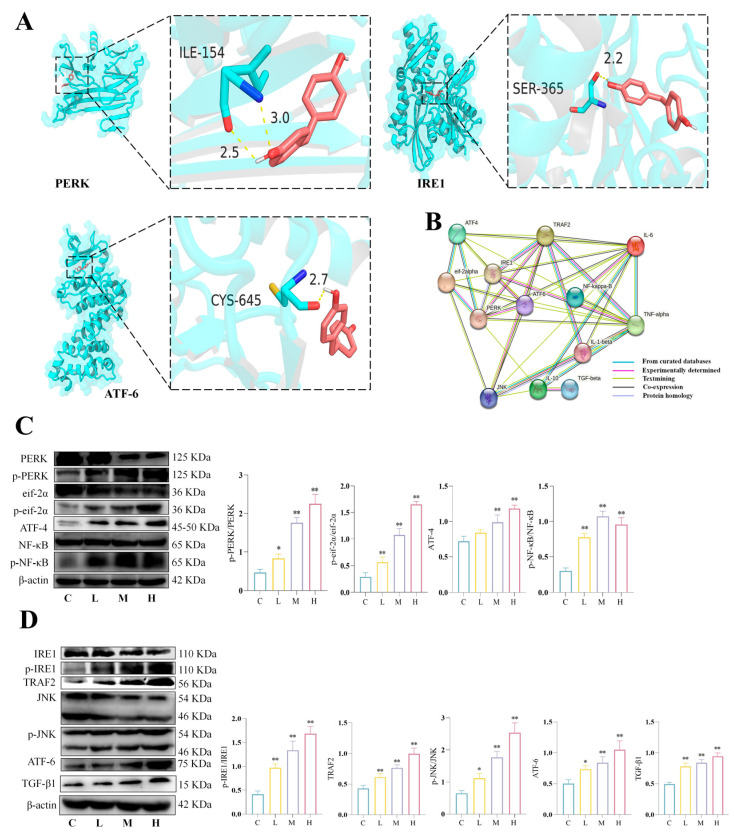
**BPF exposure activates renal ER stress, NF-κb and JNK pathways in mice**. (**A**) Molecular docking results of BPF with PERK, IRE1 and ATF-6. Hydrogen bonds are represented by yellow dashed lines, indicating specific interactions between the ligand and protein residues. Key interacting residues are labeled, and bond distances are noted. (**B**) PPI network of ER stress and inflammatory factors. (**C**) BPF activates the PERK/eif-2α/ATF-4/NF-κB signaling pathway in mice kidney (n = 3). (**D**) BPF activates the IRE1/TRAF-2/JNK and ATF-6 signaling pathway in mice kidney (n = 3). * *p* < 0.05 or ** *p* < 0.01 versus C value.

In order to investigate the mechanism by which BPF leads to macrophage polarization, molecular docking was performed with BPF and PERK, IRE1, and ATF-6, respectively (Figure 3A). The results demonstrated that BPF exhibited interactions with their residues. Specifically, BPF formed two hydrogen bonds with PERK at the ILE-154 site; one hydrogen bond with IRE1 at the SER-365 site, and one hydrogen bond with ATF-6 at the CYS-645 site. Furthermore, calculations of binding energy demonstrated that BPF demonstrated robust binding affinity for PERK, IRE1, and ATF-6, with binding energies falling below −5 kcal/mol. As demonstrated in Table S3, the binding energy of BPF docking to the target proteins is evident. Molecular docking predicted potential interactions between BPF and ER stress-related proteins, which were subsequently validated by experimental data. As shown in Figure 3B, the PPI network showed associations between ER stress, macrophage polarization, inflammation, and fibrosis. To verify whether BPF activates ER stress, we examined the protein expression of ER stress and their regulated NF-κB and JNK signaling pathways. The results revealed that BPF significantly induced an elevation in the protein expression of p-PERK/PERK, p-eukaryotic initiation factor 2α (eif-2α) /eif-2α, the activation of transcription factor 4 (ATF-4), and p-NF-κB/NF-κB (Figure 3C) (*p* < 0.01). Then, we found that BPF significantly increased the protein expression of p-IRE1/IRE1, tumor necrosis factor receptor-related factor 2 (TRAF2), p-JNK/JNK, ATF-6 and TGF-β1 (Figure 3D) (*p* < 0.01). These results indicate that BPF may mediate macrophage M1 polarization via ER stress-associated PERK/eif-2α/NF-κB signaling and macrophage M2-type polarization via ER stress-associated IRE1/TRAF2/JNK and ATF-6 signaling in the mice kidney.

### 3.4. 4-PBA Inhibits ER Stress and Macrophage M1/M2 Polarization in BPF-Exposed Raw264.7 Cells

**Figure 4:**
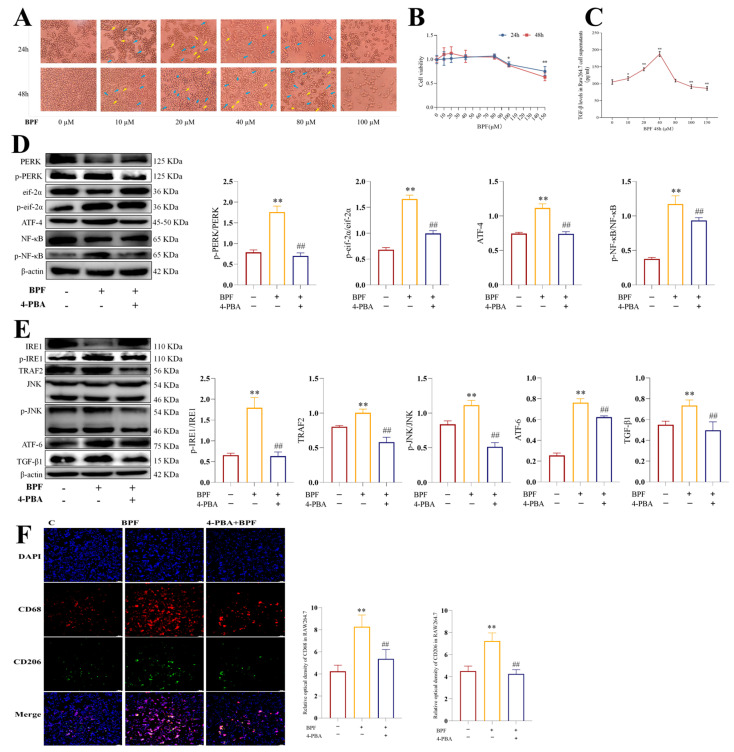
**4-PBA inhibits ER stress and macrophage M1/M2 polarization in BPF-exposed Raw264.7 cells.** (**A**) Effect of BPF on the morphology of Raw264.7 cells (n = 3), M1 phenotype (yellow arrow); M2 phenotype (blue arrow), magnification: 200×. (**B**) Effect of BPF on the viability of Raw264.7 cells (n = 4). (**C**) Effect of BPF on TGF-β1 content in the supernatant of Raw264.7 cells (n = 3). (**D**) Effect of 4-PBA on PERK/eif-2α/ATF-4/NF-κB signaling pathway-related protein expression of BPF-exposed Raw264.7 cells (n = 3). (**E**) Effect of 4-PBA on the expression of IRE1/TRAF-2/JNK and ATF-6 pathway-related proteins in BPF-exposed Raw264.7 cells (n = 3). (**F**) Effect of 4-PBA on M1/M2 polarization in BPF-exposed Raw264.7 cells (n = 3), scale: 50 µm. * *p* < 0.05 or ** *p* < 0.01 versus C value, ^#^ *p* < 0.05 or ^##^ *p* < 0.01 versus BPF value.

To further investigate the relationship between BPF-induced ER stress and macrophage polarization with renal inflammation and fibrosis in mice, we performed in vitro experiments using Raw264.7, TCMK-1, and NIH3T3 cells. Initially, the concentration of BPF that induced macrophage M1/M2 polarization was screened, and an in vitro model of BPF-exposed macrophage M1/M2 polarization was established. As demonstrated in Figure 4A, 40 µM BPF significantly induced M1/M2 polarized morphology in Raw264.7 cells. There was minimal alteration in cell density. The polarized phenotype induced by 48 h of BPF exposure manifested more distinctly than the phenotype induced by 24 h of exposure. Subsequently, the impact of BPF on Raw264.7 cell viability was investigated. It was ascertained that concentrations of BPF below 80 µM did not demonstrate a significant impact on the viability of Raw264.7 cells following 24 or 48 h exposure (Figure 4B) (*p* < 0.05 or *p* < 0.01). Concurrently, we conducted a quantitative analysis of TGF-β1 levels in the culture medium of Raw264.7 cells following 48 h exposure to different concentrations of BPF and found that 40 µM BPF had the highest levels of TGF-β1 in the culture medium of Raw264.7 cells (Figure 4C) (*p* < 0.05 or *p* < 0.01). In light of these findings, we have selected 40 µM BPF exposure for 48 h to construct an in vitro model of BPF-induced macrophage M1/M2 polarization. Furthermore, 10 µM 4-PBA was incorporated to establish a model of ER stress intervening in BPF-exposed Raw264.7 cells. As shown in Figure 4D, BPF resulted in increased protein expression levels of ER stress-related pathways regulating macrophage M1-type polarization, including p-PERK/PERK, p-eif-2α/eif-2α, ATF-4, and p-NF-κB/NF-κB, and this enhancement was found to be significantly inhibited by 4-PBA in Raw264.7 cells (*p* < 0.01); meanwhile, the BPF-induced increase in the expression of the ER stress-related pathway proteins p-IRE1/IRE1, TRAF2, p-JNK/JNK, ATF-6, and TGF-β1, which regulate macrophage M2-type polarization, was also significantly inhibited by 4-PBA (Figure 4E) (*p* < 0.05 or *p* < 0.01). The above results indicated that the ER stress intervened in the BPF-exposed Raw264.7 cell model, which was successfully constructed. Subsequent analysis revealed that BPF led to an enhancement in the fluorescence intensity of CD68 and CD206 in Raw264.7 cells. This effect was notably mitigated by the addition of 4-PBA (Figure 4F) (*p* < 0.05 or *p* < 0.01). The aforementioned findings indicated that BPF induced ER stress-related pathway activation as well as M1- and M2-type polarization in Raw264.7 cells, whereas the inhibition of ER stress significantly attenuated BPF-induced polarization. Thus, we can tentatively determine that BPF regulates M1 and M2 polarization via ER stress in Raw264.7 cells.

### 3.5. Inhibition of ER Stress in Raw264.7 Cells Attenuates BPF-Induced Inflammatory Responses as Well as MMT, EMT and FMT Processes

**Figure 5:**
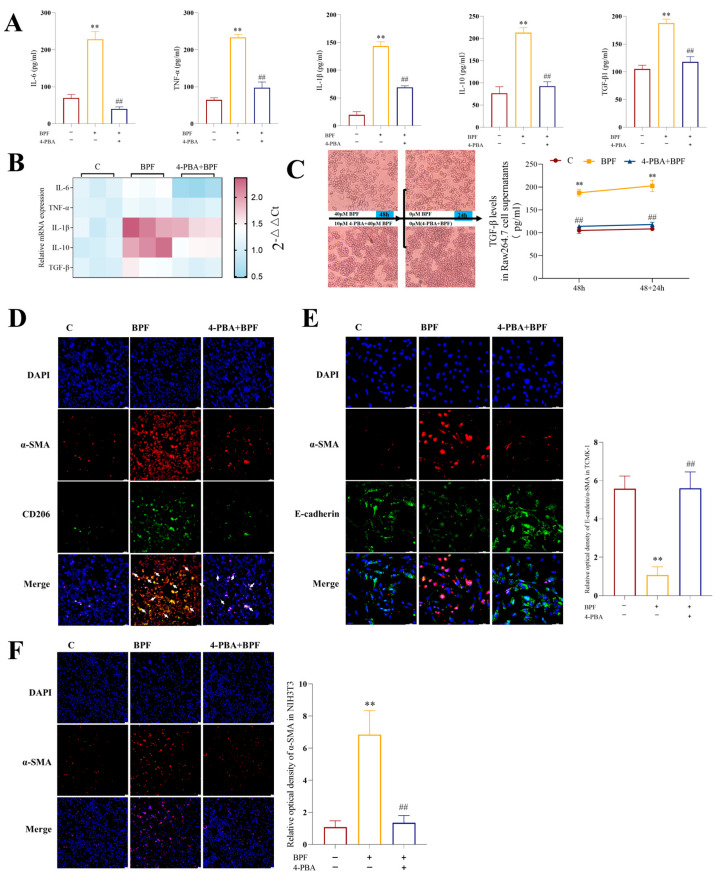
**Inhibition of ER stress attenuates BPF-induced inflammatory response in Raw264.7 cells and MMT, EMT and FMT processes.** (**A**) Effect of 4-PBA on the protein expression of inflammation-related factors in BPF-exposed Raw264.7 cells (n = 3). (**B**) Heatmap showing mRNA expression levels of renal inflammatory factors in BPF-exposed Raw264.7 cells (n = 3). (**C**) TGF-β1 content in the supernatant of BPF and 4-PBA groups of Raw264.7 cells (n = 3). (**D**) Effect of 4-PBA on MMT in BPF-exposed Raw264.7 cells (n = 3), scale: 50 µM. (**E**) Extent of the EMT of TCMK-1 cells co-cultured with 4-PBA+BPF-exposed Raw264.7 cells (n = 3), scale: 50 µm. (**F**) Extent of the FMT of NIH-3T3 cells co-cultured with 4-PBA+BPF-exposed Raw264.7 cells (n = 3), scale: 50 µm. * *p* < 0.05 or ** *p* < 0.01 versus C value, ^#^ *p* < 0.05 or ^##^ *p* < 0.01 versus BPF value.

To ascertain the impact of ER stress-mediated macrophage M1/M2 polarization on inflammation and fibrosis, an initial examination of the expression of inflammatory factors in Raw264.7 cells was conducted. The results indicated that the mRNA expression and secretion levels of IL-6, TNF-α, IL-1β, IL-10, and TGF-β1 were significantly increased in the BPF group compared with the C group. In contrast, the mRNA expression and secretion of these inflammatory factors were significantly reduced in the 4-PBA+BPF group compared with the BPF group (Figure 5A,B) (*p* < 0.01). These results suggest that BPF significantly induces increased expression of pro- and anti-inflammatory cytokines in Raw264.7 cells, which is alleviated by the inhibition of ER stress.

In order to distinguish the impact of BPF-induced macrophage polarization on MMT, EMT and FMT, while concurrently excluding the effect of BPF and 4-PBA+BPF on co-cultured cells, the Raw264.7 cell line was subjected to 48 h of exposure to BPF and 4-PBA+BPF. Subsequently, the medium was replaced with a medium devoid of BPF and 4-PBA+BPF. Following a 24 h incubation period, the quantity of TGF-β1 present in the culture medium was measured. It was ascertained that PBF-treated cells were capable of stable secretion of TGF-β1 even in the absence of BPF. Similarly, the 4-PBA+BPF group obtained the expected results (Figure 5C) (*p* < 0.01). In the subsequent phase of the study, we investigated whether Raw246.7 cells exhibited MMT in response to BPF. The findings revealed an augmented degree of co-localization of CD206 and α-SMA in the BPF group in comparison to the control group. Conversely, the incorporation of 4-PBA substantially mitigated this phenomenon induced by BPF (Figure 5D). Subsequently, we co-cultured BPF- and 4-PBA+BPF-exposed Raw264.7 cells with TCMK-1 cells and NIH3T3 cells following the replacement of the medium without the administration of any drugs. The results demonstrated that the ratio of α-SMA/E-cadherin fluorescence intensity was elevated in TCMK-1 cells and that the fluorescence intensity of α-SMA was enhanced in NIH3T3 cells under the BPF group compared with that of group C. The addition of 4-PBA significantly alleviated this phenomenon (Figure 5E,F) (*p* < 0.01). The aforementioned findings indicate that the inhibition of BPF-exposed Raw264.7 cell ER stress alleviates M1/M2 polarization and further ameliorates inflammation and fibrosis caused by BPF exposure.

### 3.6. 4-PBA Inhibits Renal ER Stress and Macrophage M1/M2 Polarization in BPF-Exposed Mice

**Figure 6:**
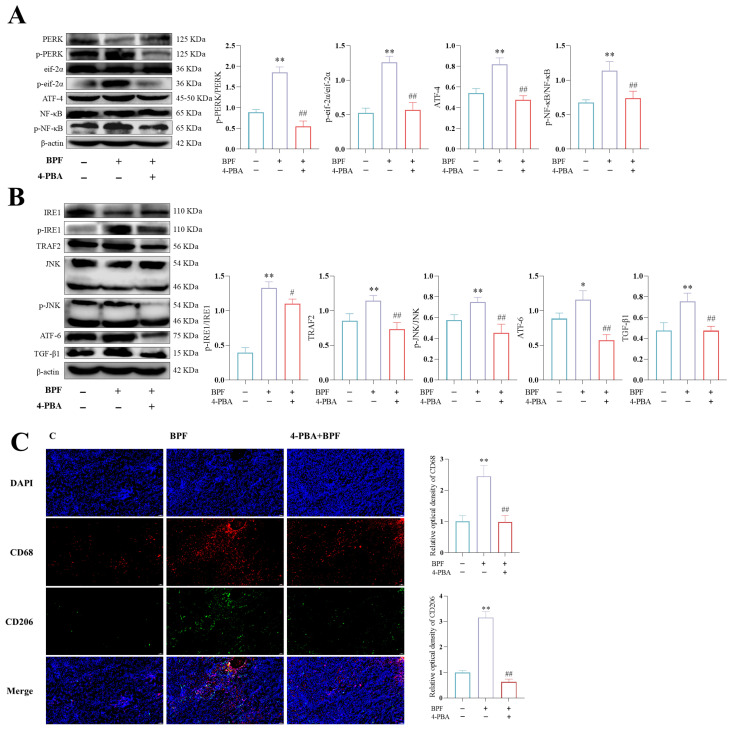
**4-PBA inhibits renal ER stress and macrophage M1/M2 polarization in BPF-exposed mice.** (**A**) Effect of 4-PBA on the kidney PERK/eif-2α/ATF-4/NF-κB signaling pathway-related protein expression in BPF-exposed mice (n = 3). (**B**) Effect of 4-PBA on the expression of IRE1/TRAF-2/JNK and ATF-6 pathway-related proteins in BPF-exposed mice kidney (n = 3). (**C**) Effect of 4-PBA on the kidney macrophage M1/M2 polarization in BPF-exposed mice (n = 3), scale: 50 µm. * *p* < 0.05 or ** *p* < 0.01 versus C value, ^#^ *p* < 0.05 or ^##^ *p* < 0.01 versus BPF value.

In order to verify that BPF-induced renal impairment is related to ER stress, a 4-PBA intervention BPF exposure mice model was established for testing. As anticipated, the incorporation of 4-PBA impeded BPF, resulting in augmented p-PERK/PERK, p-eif-2α/eif-2α, ATF-4, and p-NF-κb/NF-κb protein expression in murine kidneys (Figure 6A) (*p* < 0.01); concurrently, the augmented expression of p-IRE1/IRE1, TRAF2, p-JNK/JNK ATF-6 and TGF-β1 proteins was also significantly repressed (Figure 6B) (*p* < 0.01). Subsequent results showed that 4-PBA significantly inhibited the BPF exposure-induced elevation of CD68 and CD206 fluorescence intensities in mouse kidneys (Figure 6C) (*p* < 0.01). The aforementioned findings indicate that the inhibition of ER stress significantly alleviates BPF-induced M1/M2 polarization in mouse kidney macrophages.

### 3.7. Inhibition of ER Attenuates Kidney Inflammations and Fibrosis in BPF-Exposed Mice

**Figure 7:**
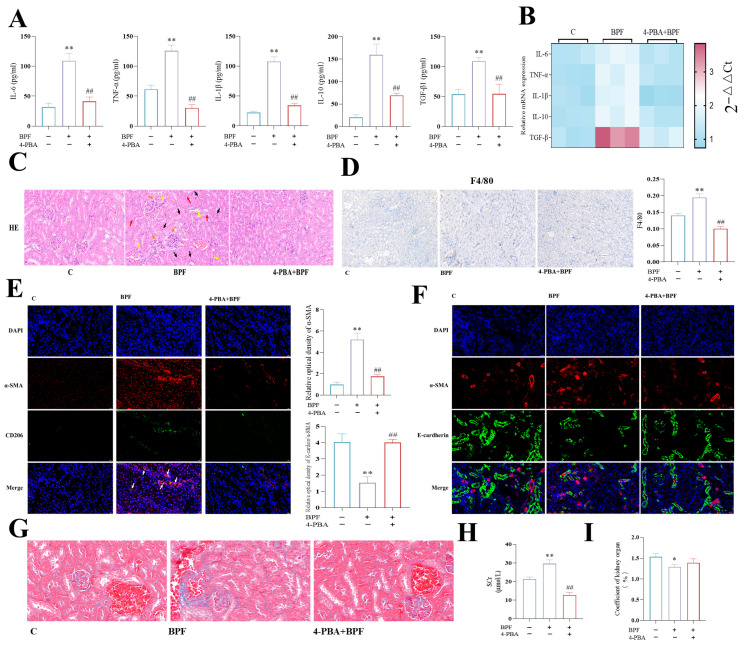
**Inhibition of ER attenuates kidney nephritis and fibrosis in BPF-exposed mice.** (**A**) Effect of 4-PBA on the protein expression of kidney inflammation-related factors in BPF-exposed mice (n = 3). (**B**) Heatmap showing mRNA expression levels of renal inflammatory factors in 4-PBA + BPF-exposed mice (n = 3). (**C**) Representative HE staining images of kidney sections from one mouse per group (n = 3), scale bar: 20 μm. Glomerular atrophy (orange arrow), tubular epithelial cell detachment (yellow arrow), blood exudation (red arrow), and inflammatory cell infiltration (black arrow) are indicated. (**D**) Effect of 4-PBA on macrophage infiltration in kidneys of BPF-exposed mice (n = 3), (F4/80) scale: 50 µm. (**E**) Effect of 4-PBA on kidney MMT and FMT in BPF-exposed mice (n = 3), scale: 20 µm. (**F**) Effect of 4-PBA on kidney EMT in BPF-exposed mice (n = 3), scale: 20 µm. (**G**) Effect of 4-PBA on renal collagen and collagen fibers in BPF-exposed mice. (**H**) Effect of 4-PBA on SCr in BPF-exposed mice (n = 3), scale: 50 µm. (**I**) Effect of 4-PBA on body weight and kidney organ coefficient in BPF-exposed mice (n = 4). * *p* < 0.05 or ** *p* < 0.01 versus C value, ^#^ *p* < 0.05 or ^##^ *p* < 0.01 versus BPF value.

In order to provide further validation that ER stress-induced macrophage polarization is a key factor in BPF-induced renal inflammation and fibrosis in mice, renal inflammation and fibrosis indices were examined in 4-PBA+BPF-exposed mice. The results demonstrated that 4-PBA significantly inhibited the increased mRNA and protein expression of renal inflammatory factors IL-6, TNF-α, IL-1β, IL-10, and TGF-β1 in mice exposed to BPF (Figure 7A,B) (*p* < 0.05 or *p* < 0.01). Concurrently, renal macrophage infiltration, tubular epithelial cell detachment, and glomerular atrophy were also significantly alleviated by 4-PBA (Figure 7C,D) (*p* < 0.05 or *p* < 0.01). In addition, the effects of BPF exposure on the ratio of E-cadherin/α-SMA fluorescence intensity, the increased co-localization of α-SMA and CD206, and the increased renal α-SMA fluorescence intensity were significantly reversed by 4-PBA in murine kidneys (Figure 7E,F) (*p* < 0.05 or *p* < 0.01). Also, the increased renal collagen fibers and collagen in the kidneys of mice were significantly reversed by 4-PBA (Figure 7G). These results suggest that the inhibition of ER stress significantly alleviated renal inflammation and EMT-, MMT-, and FMT-mediated renal fibrosis in BPF-exposed mice.

Finally, we found that 4-PBA significantly ameliorated the increase in SCr in BPF-exposed mice (Figure 7H) (*p* < 0.01). Body weight loss and renal organ coefficients in BPF-exposed mice were also ameliorated by 4-PBA (Figure 7I) (*p* < 0.01). The aforementioned findings indicate that the inhibition of ER stress significantly alleviated renal injury and insufficiency in BPF-exposed mice.

## 4. Discussion

In recent years, the prevalence of BPA alternatives (including BPS, BPF, BPZ, BPM, etc.) has raised concerns about their potential environmental impacts and human hazards [33,34]. Among these substitutes, BPF has been observed to potentially exhibit a heightened ecological risk in comparison to BPA [9]. Previous studies have shown that urinary BPF concentrations are positively correlated with human serum uric acid and urinary protein levels, and indicate an increased risk of nephropathy, suggesting that BPF exposure can lead to renal dysfunction [26,35]; however, the underlying mechanisms remain to be elucidated. In this study, we investigated the nephrotoxic effects of BPF exposure using in vivo and in vitro experimental models and explored the potential mechanisms of action. First, BPF caused renal insufficiency with renal macrophage recruitment, infiltration, increased expression of renal inflammatory factors and renal fibrosis in mice. Subsequently, it was found that these injuries might be related to BPF activation of ER stress-related pathway-mediated M1 and M2 polarization of renal macrophages in mice. Further analysis showed that the ER stress inhibitor 4-PBA significantly attenuated BPF exposure-induced M1 and M2 polarization of Raw264.7 cells, which led to a decrease in the mRNA expression of inflammatory factors in Raw264.7 cells, as well as inhibiting the MMT of Raw264.7 cells, the EMT of co-cultured TCMK-1 cells, and the FMT of co-cultured NIH3T3 cells. These results suggest that ER-mediated macrophage M1/M2 polarization may be the mechanism of the BPF-induced renal fibrosis in mice. Finally, the above hypothesis was verified by establishing an experimental model of 4-PBA intervention in BPF-exposed mice. In conclusion, the present study demonstrated that BPF causes renal dysfunction by activating ER stress to mediate macrophage M1/M2 polarization, which in turn induces renal inflammation and MMT-, EMT-, and FMT-mediated renal fibrosis in mice.

SCr is one of the important indicators to measure renal function [36]. Creatinine is a product of muscle metabolism and is excreted mainly through glomerular filtration. When glomerular filtration rate (GFR) decreases, SCr levels increase, suggesting impaired renal function [37]. Our results showed that BPF led to increased SCr levels in mice, indicating that BPF can lead to a decrease in GFR and cause renal dysfunction. Fibrosis is an important contributor to renal dysfunction caused by exogenous toxicants [38]. In the process of renal fibrosis, pathological changes such as tubulointerstitial damage, excessive deposition of extracellular matrix, and glomerulosclerosis will destroy the normal structure of the kidney [39]. These structural changes have a direct impact on glomerular filtration and are the direct cause of kidney failure [40]. Inflammation is closely related to fibrosis and is an important initiator and driver of fibrosis [41]. Renal inflammation can lead to the release of a variety of cytokines and inflammatory mediators, which activate renal intrinsic cells and fibroblasts, promote excessive deposition of extracellular matrix, and ultimately lead to renal fibrosis [42]. Meanwhile, renal fibrosis also triggers vascular injury and inflammatory response, further aggravating renal dysfunction and forming a vicious cycle [43]. Our results showed that BPF caused renal collagen fiber and collagen deposition as well as glomerular atrophy, detachment of tubular epithelial cells, increased renal hemorrhagic spots, and inflammatory cell infiltration. These suggest that BPF-induced renal failure is associated with renal fibrosis and inflammation.

M1/M2 polarization of macrophages plays a key role in nephritis and renal fibrosis. Changes in their polarization status directly affect the renal inflammatory response and fibrosis process [44]. At one extreme, the M1 pro-inflammatory cell phenotype contributes to the clearance of infection but also secretes pro-inflammatory cytokines (e.g., IL-6, TNF-α, and IL-1β) to promote the renal inflammatory response [45]. On the other extreme, M2 anti-inflammatory cells have a reparative phenotype that secretes the anti-inflammatory cytokines (e.g., IL-10 and TGF-β1) to promote the ablative phase of the injury response [46,47]; however, this phenotype also promotes fibrosis, which is a major driver of progression to end-stage renal disease [44]. Our results showed that BPF led to increased mRNA and protein expression of pro- and anti-inflammatory cytokines, and induced renal macrophage recruitment and infiltration as well as increased CD68 and CD206 fluorescence intensity. In addition, BPF-exposed Raw264.7 cells showed increased anti- and pro-inflammatory cytokines as well as CD68 and CD206 fluorescence intensities. These results suggest that BPF may induce renal inflammatory response and fibrosis by causing renal macrophage recruitment and its polarization toward M1 and M2 phenotypes to secrete inflammatory cytokines.

Numerous studies have demonstrated that the CD206 subpopulation of M2 macrophages is strongly associated with human renal fibrosis and experimental disease [48,49]. This is closely related to the fact that TGF-β1, an anti-inflammatory cytokine secreted by M2 macrophages, promotes the transformation of renal cell populations into myofibroblasts [50]. Myofibroblasts play a pivotal role in kidney fibrosis and are characterized by the expression of α-SMA, the main cell type involved in the formation of fibrotic collagen matrix [51]. In renal fibrosis, myofibroblasts may originate from perivascular fibroblasts [51], macrophages [52], and renal tubular epithelial cells [53]. TGF-β1 secreted by M2-type macrophages can be bound by the TGF-β1 receptor on the surface of renal tubular epithelial cells, leading to EMT via the TGF-β1/Smad3 pathway [54]. Meanwhile, TGF-β1 can activate the TGF-β1/Smad3 pathway in M2 macrophages themselves to mediate the MMT process [55]. In addition, TGF-β1 activates the fibroblast JAK/STAT3 pathway, which in turn promotes FMT [56]. That is, TGF-β1 secreted by M2 macrophages mediates FMT in perivascular fibroblasts, MMT in M2-type macrophages, and EMT in renal tubular epithelial cells as the main drivers of renal fibrosis. Our results showed that BPF resulted in increased renal TGF-β1 protein expression in mice. BPF also caused enhanced renal α-SMA fluorescence and perivascular collagen deposition, increased co-localization of renal α-SMA with CD206, and decreased peritubular E-cadherin/α-SMA fluorescence intensity ratio in mice. Similarly, BPF caused an increase in TGF-β1 content in the supernatant of Raw264.7 cells, an increase in the co-localization of α-SMA with CD206 in Raw264.7 cells, an enhancement of α-SMA fluorescence signals in NIH3T3 cells and a decrease in E-cadherin/α-SMA fluorescence intensity ratio in the TCMK-1 cells that are co-cultured with BPF-exposed Raw264.7 cells. The aforementioned results suggest that BPF induces the polarization of the macrophages to the M2 type, which leads to renal MMT, EMT, and FMT, resulting in renal fibrosis. However, the specific mechanism by which BPF induces polarization in renal macrophages remains to be elucidated.

The ER, a critical organelle, plays a pivotal role in facilitating a multitude of essential cellular processes, including protein synthesis, folding, modification, and the maintenance of calcium ion homeostasis [57]. ER disruptions result in the accumulation of unfolded or misfolded proteins, which, in turn, activate a series of cellular stress responses. ER stress has been demonstrated to play a pivotal role in the pathophysiology of inflammation and fibrosis [57]. It contributes to the development of inflammatory response and fibrosis through various mechanisms [58]. In recent years, ER stress has been recognized as a key signaling pathway in response to BPF [59,60]. Furthermore, the process of ER stress regulates the polarization of macrophages through multiple signaling pathways. This process plays an important role in a wide range of diseases, including inflammation, tissue repair, and fibrosis [61,62]. PERK, IRE1 and ATF-6 are transmembrane proteins located on the ER that respond to ER stress caused by various stimuli [58]. The molecular docking results showed that BPF could bind to its amino acid residues through hydrogen bonding, suggesting that ER stress is most likely the mechanism by which BPF leads to the polarization of renal macrophages. Meanwhile, the PPI results further illustrated the interaction between ER stress and macrophage polarization, inflammation and fibrosis. Under ER stress conditions, PERK is activated to phosphorylate eIF2α, which inhibits overall protein synthesis but promotes ATF4 translation. ATF-4 is a nuclear transcription factor that activates NF-κB and enables nuclear translocation [63]. The activation of the NF-κB signaling pathway has been shown to promote the transcription of pro-inflammatory cytokines, to mediate the polarization of macrophages towards the M1 type, and to enhance their inflammatory response [64,65]. The present study demonstrated that BPF induced an augmentation in the protein expression of p-PERK/PERK, p-eif-2α/eif-2α, ATF-4, and p-NF-κB/NF-κB in murine kidneys and Raw264.7 cells. This finding indicates that BPF may play a role in regulating macrophage polarization towards the M1 type by modulating NF-κB activity through the ER-associated PERK/eif-2α/ATF-4 signaling pathway. IRE1α is an ER transmembrane protein containing serine/threonine kinase and endonuclease structural domains. Under ER stress conditions, the activation of IRE1α generates a binding site for TRAF2, which upon binding can further activate JNK [66]. The activation of JNK further regulates cellular stress and inflammatory responses. In certain inflammatory responses, the activation of JNK has been observed to affect the transcription of IL-10 and TGF-β1 by regulating the activity of transcription factors [67]. Furthermore, under specific pathological conditions, the activation of JNK has been observed to enhance TGF-β1 secretion, thus promoting fibrosis and immunosuppression [62,68]. ATF-6 is a transcription factor localized in the ER and belongs to the leucine zipper transcription factor family [69]. In typical circumstances, ATF-6 attaches to the ER molecular chaperone GRP78, thereby becoming firmly embedded within the ER. In circumstances of ER stress, the separation of GRP78 from ATF-6 occurs, and ATF-6 is subsequently translocated to the Golgi apparatus. There, it undergoes cleavage by S1P and S2P proteases, resulting in the release of the N-terminal 50 kD cytoplasmic portion, which contains nuclear localization sequences. This portion enters the nucleus, where it functions as a transcription factor [70]. In immune cells, ER stress-activated ATF-6 enhances TGF-β1 secretion, which promotes macrophage polarization toward the M2 type, suppresses inflammatory responses and promotes fibrotic processes [71]. Our results showed that BPF caused an increase in p-IRE1/IRE1, TRAF2, ATF-6 and p-JNK/JNK protein expression in mice kidney and Raw264.7 cells. This finding indicates that BPF may play a regulatory role in the polarization of macrophages toward the M2 type by modulating the secretion of TGF-β through the ER-associated IRE1/TRAF2/JNK and ATF-6 signaling pathway. The collective impact of ER stress-mediated M1 and M2 polarization of macrophages may serve as the underlying mechanism of BPF-induced renal fibrosis in mice.

To investigate whether ER stress-mediated macrophage polarization is the cause of BPF-induced renal fibrosis in mice, we treated BPF-exposed Raw264.7 cells with the ER stress inhibitor 4-PBA to establish a model of in vitro ER stress intervention in BPF-exposed macrophage polarization. The results showed that 4-PBA treatment significantly ameliorated the BPF-induced activation of ER stress-related signaling pathways and macrophage M1/M2 polarization in Raw264.7 cells. Concurrently, the augmentation in anti-inflammatory and pro-inflammatory cytokine transcription, along with the escalation in superantigen TGF-β1 content, were demonstrably repressed by 4-PBA in BPF-exposed Raw264.7 cells. Furthermore, the MMT process was found to be significantly inhibited in 4-PBA-intervened BPF-exposed Raw264.7 cells, and the FMT of NIH3T3 cells and EMT of TCMK-1 cells co-cultured with them were also improved. The findings indicate that the suppression of macrophage ER stress is a probable mechanism for mitigating renal fibrosis in mice exposed to BPF. To validate this finding, a 4-PBA intervention was conducted in mice exposed to BPF. As anticipated, the suppression of ER stress led to a substantial alleviation of renal macrophage M1/M2 polarization, inflammation, and fibrosis in mice exposed to BPF, consequently reducing the elevated SCr levels.

This study has the following limitations. First, although the low dose (0.5 mg/kg) was determined based on human biomonitoring data and interspecies conversion, the medium and high doses (5 and 50 mg/kg) exceed typical environmental exposure levels. This dose regimen facilitates the identification of toxic mechanisms within a limited experimental timeline; therefore, the findings should be regarded as mechanistic discoveries rather than direct inferences about human risk. Second, while we comprehensively assessed macrophage polarization using CD68, CD206, and cytokine profiles, CD68 is a pan-macrophage marker rather than an M1-specific indicator. Future studies should incorporate M1 markers like CD86 and iNOS alongside M2 markers such as CD163 and Arg-1, combined with flow cytometry for more precise phenotypic characterization. Furthermore, this study only examined the effects of 28-day exposure. Future research should investigate chronic, low-dose exposure to better simulate real-world scenarios and assess long-term renal injury risks. In addition to inhibiting ER stress, 4-PBA exhibits other biological functions such as histone deacetylase inhibition and anti-inflammatory effects. Therefore, the observed protective effects cannot be entirely attributed to the suppression of ER stress. In future studies, we will employ more specific approaches, such as macrophage-specific PERK or IRE1 knockout mice, or combine RNA interference techniques, to further confirm the pivotal role of ER stress in mediating BPF nephrotoxicity across different cell types. In summary, despite these limitations, the consistent findings across multiple model systems provide reliable evidence for the key role of ER stress-mediated macrophage polarization in BPF-induced nephrotoxicity.

## 5. Conclusions

This study demonstrates that BPF exposure is associated with renal ER stress activation, macrophage M1/M2 polarization, inflammation, and fibrosis. The data support a model in which ER stress-mediated macrophage polarization may contribute to BPF-induced nephrotoxicity, though further studies are needed to establish definitive causality.

## Data Availability

The original contributions presented in this study are included in the article/Supplementary Material. Further inquiries can be directed to the corresponding authors.

## Supplementary Materials

The following supporting information can be downloaded at: https://www.mdpi.com/article/10.3390/toxics14030255/s1, Text S1: Masson staining; Text S2: ELISA; Text S3: Immunofluorescence; Text S4: qRT-PCR; Table S1: Antibody Information; Table S2: Primers Used for Quantitative Real-time PCR; Table S3. Binding energy of BPF docking to the target proteins.

## Abbreviations

The following abbreviations are used in this manuscript:

BPF	Bisphenol F
4-PBA	4-Phenylbutyric acid
ER	endoplasmic reticulum
MMT	macrophage–myofibroblast transformation
EMT	epithelial–mesenchymal transition
FMT	fibroblast–myofibroblast transformation
BPA	Bisphenol A
SCr	serum creatinine
HE	Hematoxylin-eosin
eif-2α	eukaryotic initiation factor 2α
ATF-4	activation of transcription factor 4
PERK	protein kinase R-like endoplasmic reticulum kinase
IRE1	inositol-requiring enzyme 1
PPI	protein–protein interaction
TRAF2	tumor necrosis factor receptor-related factor 2
ATF-6	activation of transcription factor 6
