# Supplementary material for: Bisphenol F Drives Endoplasmic Reticulum Stress-Mediated Macrophage Polarization, Leading to Inflammation and Fibrosis in Mouse Kidneys
Source: Toxics. 2026 Mar 13;14(3):255. doi: 10.3390/toxics14030255 (PMC13030847; doi:10.3390/toxics14030255)
Supplement: Supplementary file 1 [file toxics-14-00255-s001.zip › toxics-4132649-supplementary.pdf]

Bisphenol F Drives Endoplasmic Reticulum Stress-Mediated Macrophage Polarization, Leading to Inflammation and Fibrosis in Mouse Kidneys

Chenjiao Miao [a, 1], Yang Fu [a, 1], Binwen Zhang [a], Wangyong Yu [a], Miao Song [a], Yanfei Li [a]*, Zheng Cao [a]*

a. Heilongjiang Key Laboratory for Laboratory Animals and Comparative Medicine, College of Veterinary Medicine, Northeast Agricultural University, Harbin, 150030, China.

*. Corresponding author. College of Veterinary Medicine, Northeast Agricultural University, NO. 600, Changjiang Road, Harbin, 150030, China. E-mail address: yanfeili_200@126.com (Yanfei Li), neaucz@163.com (Zheng. Cao).

1. The authors contributed equally to this study.

Text S1. Masson staining:

Kidney tissues were fixed using pre-cooled formaldehyde at 4°C and samples were sent to Sevier Biotechnology Ltd. for staining. Following deparaffinization of the paraffin sections, the nuclei were stained with Regaud hematoxylin for 8 min, followed by 1% hydrochloric acid alcohol differentiation for 10 s. Subsequently, 1% lithium carbonate was applied for 5 min to enhance the anti-blue effect, and Masson Lichun Red Acid Reducing Solution was used for an additional 5-10 min. The sections were then treated with 2% aqueous glacial acetic acid for 1 minute. Next, differentiation was carried out using a 1% aqueous phosphomolybdic acid solution for 3-5 min, followed by staining with aniline blue aqueous solution or 1% light green aqueous solution for 1-2 min. Following the application of a 0.2% aqueous glacial acetic acid solution, the specimen was subjected to a dehydration process using 95% ethanol. This was followed by three cycles of dehydration in anhydrous ethanol, with each cycle lasting for a duration of five seconds. The sections were subsequently subjected to a series of xylene baths, each lasting 1 min, before being sealed in neutral gum.

Text S2. ELISA

Kidney tissues were homogenized in ice-cold PBS (100 mg tissue/mL) containing protease inhibitor cocktail (Roche, Switzerland). Homogenates were centrifuged at 12,000 × g for 15 min at 4°C, and supernatants were collected. Protein concentrations in tissue homogenates were determined using a BCA Protein Assay Kit (Beyotime, Shanghai, China) and used to normalize cytokine levels (expressed as pg/mg protein). For cell culture experiments, supernatants from Raw264.7 cells were collected and

centrifuged at 1000 × g for 10 min at 4°C to remove debris.

The concentrations of IL-6, TNF-α, IL-1β, IL-10, and TGF-β1 were quantified using commercially available ELISA kits (IL-6, TNF-α, IL-1β, IL-10: JONLNBIO, Shanghai, China; TGF-β1: JINGMEIBIO, Jiangsu, China) according to the manufacturers' protocols. Briefly, 50 μL of standard or sample was added to each well pre-coated with capture antibody, followed by incubation at 37°C for 30 min. After washing five times with wash buffer, 50 μL of biotin-labeled detection antibody was added and incubated for 30 min at 37°C. Following another wash step, 50 μL of HRP-conjugated streptavidin was added and incubated for 15 min at 37°C. After a final wash, 50 μL of TMB substrate solution was added and incubated for 15 min at 37°C in the dark. The reaction was stopped by adding 50 μL of stop solution, and absorbance was measured at 450 nm using a microplate reader (BioTek, USA). All samples were assayed in duplicate. The detection limits, as provided by the manufacturers, were: IL-6: 1.0 pg/mL; TNF-α: 2.0 pg/mL; IL-1β: 1.5 pg/mL; IL-10: 1.0 pg/mL; TGF-β1: 1.0 pg/mL.

Text S3. Immunofluorescence.

Kidney tissues were fixed using pre-cooled formaldehyde at 4°C and samples were sent to Sevier Biotechnology Ltd. for staining. Briefly, paraffin sections were deparaffinized and hydrated, and antigen repair was performed using EDTA antigen repair solution (PH 9.0) (Servicebio, Wuhan, China). The sections were then blocked with 3% BSA (Servicebio, Wuhan, China) for 30 min and incubated with the first primary antibody in a wet box at 4°C overnight. The slides were washed three times in

PBS (PH7.4) on a decolorization shaker for 5 min each time, the sections were gently shaken dry, and the corresponding HRP-labeled secondary antibody was added dropwise to the circle and incubated at room temperature for 50 min. After washing in PBS, the corresponding TSA dye was added and incubated away from light for 10 min. Finally, the nuclei were re-stained with DAPI, and the images were captured by orthogonal fluorescence microscope (NIKON, NIKON ECLIPSE C1) and scanner (3DHISTECH, Pannoramic MIDI) after sealing with anti-fluorescence burst sealer.

Cells were inoculated at the indicated densities into laser confocal dishes or 96-well plates or Transwells for 24 h and then processed according to different groupings. After formaldehyde fixation, the plates were blocked with PBS solution containing 5% goat serum and 3% BSA for 1 h. Then, primary antibodies were added overnight at 4°C and fluorescent secondary antibodies were added and incubated for 1 h in the dark; the plates were re-stained with DAPI for 10 min, blocked with anti-fluorescence bursting agent, and then images were captured using an inverted fluorescence microscope. The corresponding antibody information is shown in Table S1.

**primary antibody name**	**primary antibody number**	**primary antibody brands**	**Dilution ratio**	**Secondary antibody name**	**Corresponding TSA**
**CD68**	GB113109		1:2000		CY3-Tyramide （tsa） /
**CD206**	GB113497	Servicebio		HRP labeled goat anti-rabbit IgG (Servicebio)	iF488-Tyramide （tsa）
**a-SMA**	GB111364		1:8000		CY3-Tyramide （tsa

**E-cadherin**	GB12083		1:6000	HRP-labeled goat anti-mouse IgG ((Servicebio)	iF488-Tyramide （tsa）
**CD68**	WL04839				
**a-SMA**	WL02510	Wan Lei	1:200	Goat Anti-rabbit IgG H＆L Alexa Flour 594)(1:1000, Bioss, Beijing, China)	
**Fibronection 1**	WL00712				
**Collage1/3**	14695-1-AP	proteintech	1:500		
**CD206**	SC-58986	SANTA	1:500	Goat Anti-mouse IgG H＆L Alexa Flour 488)(1:1000, Bioss, Beijing, China)	
**E-cadherin**	SC-8426				
**NF-κB**	WL01980				
**p-NF-κB**	WL02169				
**JNK**	WL01295				
**p-JNK**	WL01813				
**TGF-β1**	WL02193				
**PERK**	WL03378	Wan Lei			
**p-PERK**	WL05295		1:1000		
**IRE1**	WL02562			goat anti-rabbit IgG	
**p-IRE1**	WL05299				
**ATF-4**	WL02330				
**TRAF2**	WL02846				
**ATF-6**	WL01153				
**p-eif-2α**	AF3087	Affinity			
**β-actin**	AC026	Abclonal	1:10000		
**eif-2α**	sc-133132	Santa	1:1000	goat anti-mouse IgG	

Table S1. Antibody Information

Text S4. qRT-PCR.

The total RNA of jejunal tissues and IPEC-J2 cells was extracted with TRIzol® reagent (Invitrogen, CA, USA). RNA concentration in extracts was measured by Nanophotometer (IMPLEN, DE) with purity ascertained by A260/A280. The cDNA was generated from 1 μg of total RNA using PrimeScript RT reagent kit (Vazyme, Nanjing, China) and diluted to 10-fold in Rnase free water. The products were amplified using SYBR green 2×RT-PCR mix (Vazyme, Nanjing, China) and quantitative real-time PCR (qRT-PCR). The results of qRT-PCR normalized comparisons by GADPH.

GENE	Forward and reverse primer sequence
GADPH	Forward:5′-ATGGTGAAGGTCGGAGTG-3′
	Reverse: 5′-GTAGTGGAGGTCAATGAAGG-3′
IL-6	Forward:5'-CTGCAAGAGACTTCCATCCAG-3'
	Reverse:5′- AGTGGTATAGACAGGTCTGTTGG -3′
TNF-α	Forward:5′- GATGGGTTGTACCTTGTCTACT′
	Reverse: 5′- CTTTCTCCTGGTATGAGATAGC -3′
IL-1β	Forward: 5′- TTCAGGCAGGCAGTATCACTC -3′
	Reverse: 5′- GAAGGTCCACGGGAAAGACAC -3′
IL-10	Forward: 5′- CAGGGATCTTAGCTAACGGAAA -3′
	Reverse: 5′- GCTCAGTGAATAAATAGAATGGGAAC -3′
TGF-β1	Forward: 5′- TGGAGCAACATGTGGAACTC -3′
	Reverse: 5′- GTCAGCAGCCGGTTACCA -3′

Table S2. Primers Used for Quantitative Real-time PCR

Target name	Binding energy (kcal/mol)
PERK	-6.170
IRE1	-6.545
ATF-6	-6.981

Table S3. Binding energy of BPF docking to the target proteins.
